# Selective Activation of the Subscapularis Muscle: A Cross-Sectional Observational Study

**DOI:** 10.3390/healthcare13111349

**Published:** 2025-06-05

**Authors:** Donghyun Kim, Soonjee Park

**Affiliations:** 1 Institute of Human Ecology, Yeungnam University, Gyeongsan 38541, Republic of Korea; rlagus@yu.ac.kr; 2 Department of Clothing and Fashion, Yeungnam University, Gyeongsan 38541, Republic of Korea

**Keywords:** subscapularis, selective strengthening, shoulder internal rotation exercise, PICR

## Abstract

Background/Objectives: Clinicians have employed various therapeutic exercises to enhance the function and strength of the subscapularis muscle (SSC). However, few studies have investigated the most effective exercise for selectively activating the SSC while minimizing compensation from surrounding shoulder musculatures. Methods: Forty healthy participants without any shoulder complex conditions participated in this study. Individuals with a history of shoulder pain or musculoskeletal or neurological conditions affecting shoulder internal rotation were excluded. Participants performed three exercises: (1) Belly Press, (2) Lift Off, and (3) Prone Wiper, in a randomized order generated using Microsoft Excel. Ultrasound was then performed to assess the SSC and infraspinatus (IS) muscle thickness. Surface electromyography was used to record anterior deltoid (AD), pectoralis major (PM), and posterior deltoid (PD) muscle activity. Radiographic imaging was employed to evaluate the path of the instantaneous center of rotation (PICR). Data were analyzed using a one-way repeated-measures analysis of variance (ANOVA), followed by Bonferroni adjustment. Results: A significant reduction in IS thickness and PICR was observed only following the Belly Press (*p* < 0.05). Electromyographic activity of AD, PM, and PD increased significantly across all exercises (*p* < 0.05). These results demonstrate that the Belly Press most effectively allows the SSC to generate force while maintaining a stable center of rotation during shoulder movement. Conclusions: The Belly Press was most effective in selectively activating the SSC while minimizing surrounding shoulder rotator muscle activity and reducing PICR. This finding may help clinicians identify and treat patients with shoulder internal rotation injuries.

## 1. Introduction

The rotator cuff (RC) is a critical musculotendinous structure that reinforces the glenohumeral joint capsule and plays an essential role in facilitating normal shoulder movement. Because of its proximity to the shoulder joint, the RC moves in coordination with the joint and can assist in preventing joint translation [1]. Among the RC muscles, the subscapularis (SSC) functions as a powerful internal rotator and stabilizer of the shoulder joint, exerting a compressive force that positions the humeral head within the glenoid fossa during shoulder movements [2]. Additionally, the SSC exerts a downward force on the humeral head, counteracting the upward pull generated by the deltoid muscle contraction during shoulder abduction [3], and exerts forward pressure on the humeral head to prevent translation caused by extensors during shoulder extension [4,5].

SSC weakness or injury is commonly observed in athletes and non-athletes, particularly in individuals who frequently perform repetitive overhead arm movements [6,7]. During such movements, the overall arc of the shoulder joint shifts posteriorly to increase the range of external rotation, causing the greater tuberosity to be pulled further beyond the glenoid fossa, thereby causing anterior capsular laxity and reducing the internal rotation range [8,9]. Previous studies reported that anterior instability is the most significant contributor to internal impingement during shoulder internal rotation [6,10]. Fares et al. (2023) reported that the incidence of anterior shoulder instability is estimated at 0.08 cases per 1000 person–years in the general population, with a markedly higher risk of up to 3% per year among young males [11]. This type of instability is commonly associated with pain, abnormal movement patterns, and decreased functional capacity [11]. Van Iersel et al. (2023) reported that 74% of individuals with anterior shoulder instability were unable to resume sports participation, primarily due to fear of reinjury and anxiety related to physical activity [6]. Therefore, it is necessary to find the most effective exercise method and provide evidence to restore and maintain normal shoulder internal rotation function [12].

Researchers have evaluated the function and strength impairments of the shoulder internal rotation muscles using various tests. The Belly Press and Lift Off tests are commonly used assessments for SSC, and therapeutic exercises based on these two evaluation methods have been shown to significantly strengthen the SSC [13,14]. Some studies have also shown that internal rotation range of motion training during rehabilitation after the RC surgery strengthens the SSC [15,16]. Fritz et al. (2017) demonstrated daily shoulder rotation exercises following the RC surgery increased RC muscle strength, including the SSC, and improved rotation range, as assessed via 3D motion analysis and surface electromyography (EMG) [17]. Meanwhile, a previous study investigating clinical tests for evaluating the SSC function has reported that the Bear Hug test has the highest positive predictive value, and when combined with the Belly Press test, it may represent the most effective combination for diagnosing SSC pathology [18]. Another study compared the specificity and sensitivity of several diagnostic tests for SSC tears, including the Bear Hug, Belly Press, Internal Rotation Lag Sign, and Lift Off tests [19]. The study has reported that although all tests demonstrated pooled specificities greater than 0.90, their pooled sensitivities were below 0.60; therefore, no single clinical test can be considered sufficiently reliable for diagnosing SSC tears. A study comparing the effectiveness of the Belly Press, Bear Hug, and Side-lying Wiper exercises for strengthening the SSC reported that the Side-lying Wiper was the most effective in enhancing SSC activation and strength [20].

Unlike EMG, ultrasound can measure changes in muscle contraction by assessing soft tissue thickness, and its application to RC assessment is well known [21,22]. Smith et al. (2011) reported that the sensitivity of ultrasound in detecting full-thickness rotator cuff tears ranged from 92.4% to 96%, while the specificity ranged from 93.0% to 94.4% [22]. For partial-thickness tears, the sensitivity ranged from 66.7% to 84%, and the specificity ranged from 89% to 93.5% [22]. Researchers also have investigated ultrasound-based techniques for probing RC muscles and surrounding musculature from multiple angles [23] and for monitoring post-operative muscle recovery [24]. A previous study compared changes in the SSC thickness during internal rotation movements in the Belly Press, Lift Off, and prone positions and reported that the greatest increase occurred during the Belly Press [25].

According to Sahrmann, the movement of any joint creates a path of instantaneous center of rotation (PICR) [26]. When the PICR is minimized, stability and normal movement are provided to the joint. A previous study reported that the minimized PICR of the glenohumeral joint during shoulder external rotation was achieved through activation of the infraspinatus muscle (IS), which acts as both a stabilizer and a prime mover for shoulder external rotation. As a result, the posterior deltoid muscle (PD) was able to perform shoulder external rotation more stably and generate a large torque [27]. In a study aimed at strengthening the shoulder external rotation muscles in patients with shoulder impingement syndrome, Park (2018) reported that precise joint movement could occur when the center of rotation of the glenohumeral joint was maintained in the same position during arm movement [28]. However, few studies have investigated the influence of the SSC on PICR as a stabilizer and prime mover during shoulder internal rotation.

Although various SSC exercises have been studied, there is insufficient evidence regarding which exercises can selectively strengthen the SSC compared to other shoulder rotator muscles. Therefore, this study aimed to compare the effects of the SSC strengthening exercises and to determine which exercise most effectively activates the SSC selectively. The SSC exercises were Lift Off, Belly Press, and Prone Wiper. We have developed Prone Wiper, which was performed with the shoulder and elbow flexed to 90° in the prone position. Performing exercises in a prone position with the shoulder abducted to 90° simulates the biomechanical conditions encountered during athletic activities, including joint orientation, capsular strain, and optimal length–tension relationships of the muscle fibers. This position facilitates strength development while allowing the glenohumeral and scapulothoracic muscle groups to coordinate joint movement and maintain dynamic stability [29,30]. This study measured changes in the activation of the SSC and IS using ultrasound, as these muscles are not easily measured with surface EMG. The activity of superficial shoulder rotator muscles was measured using surface EMG and compared to SSC and IS activation. We also analyzed PICR during shoulder internal rotation using diagnostic imaging equipment to identify the relationship between PICR and muscle activation patterns.

## 2. Materials and Methods

### 2.1. Participants

We used G*Power version 3.1.9.2 software to calculate the required sample size. A minimum of 28 participants was required to attain an α level of 0.05 and a statistical power of 0.8 [31]. A total of 40 participants (20 males, 20 females, age = 38.95 ± 3.32 years, height = 166.447 ± 6.72 cm, weight = 65.55 ± 11.38 kg) without any shoulder complex conditions were recruited for this study. The specific inclusion criteria for participants were as follows: (1) no history of surgical procedures involving the shoulder, (2) no history of shoulder pain within the past three years, (3) no participation in any shoulder muscle strengthening program within the past six months, and (4) full shoulder range of motion as assessed by a licensed physical therapist. Individuals with a history of shoulder pain or musculoskeletal or neurological conditions affecting shoulder internal rotation were excluded. Participants were directly recruited from the general population engaged in daily activities. The purpose, procedures, and schedule of this experiment were explained, and voluntary participation was encouraged through public announcements. Each participant took part in this experiment only once, and the session was expected to last approximately 90 min. This experiment was conducted at the Physical Therapy Center and Radiology Department of S Neurosurgery Clinic in Daegu, South Korea. All participants read and signed the university-approved human subjects consent form. This study was approved by the Daegu University Institutional Review Board (1040621-201901-HR-009-02).

### 2.2. Ultrasonography

Diagnostic ultrasound is a device that utilizes high-frequency sound waves to observe soft tissues in real-time. It is particularly advantageous for assessing the activation of deep muscles, which cannot be measured using surface EMG, and allows for evaluation during dynamic movements. Given these strengths, this study employed ultrasound to measure changes in the thickness of the SSC—which are difficult to assess via surface EMG—during internal shoulder rotation. In addition, changes in the thickness of the antagonistic muscle, the IS, were also measured. However, ultrasound imaging is highly dependent on the examiner’s proficiency in handling the probe and their understanding of the anatomical and physiological characteristics of the target tissue. To ensure measurement reliability, all ultrasound assessments in this study were performed by a clinician with over 10 years of experience, well-versed in both ultrasound imaging and musculoskeletal anatomy.

A diagnostic ultrasound system (ACCUVIX V10, Samsung Medison, Seoul, Republic of Korea) equipped with a 6–12 MHz broadband linear probe (L5-13IS) was used to measure the muscle thickness of the SSC and IS. To measure SSC thickness, the probe was positioned on the lesser tubercle of the humerus at the beginning of each exercise. For IS thickness, the probe was positioned on the infraspinous fossa, approximately 4 cm below the scapular spine, and aligned parallel to it [23,24]. The participants performed each exercise with the probe in a fixed position. The criteria for measuring muscle thickness were defined as follows: for the SSC, the distance between the highest point of the lesser tubercle and the lowest point of the fascia overlying the SSC; for the IS, the distance between the highest point of the superior fascia of the IS and the highest point of the infraspinous fossa [23].

### 2.3. Surface Electromyography

Surface EMG is a non-invasive technique used to measure the electrical signals generated by muscles. It is widely utilized to assess muscle function and detect potential nerve damage. Due to its non-invasive nature, surface EMG does not cause discomfort or pain to the participants. In this study, surface EMG was employed to measure the muscle activity of the anterior deltoid (AD), pectoralis major (PM), and PD. However, unlike diagnostic ultrasound, surface EMG does not provide direct visualization of anatomical structures at the measurement site, and it is susceptible to signal noise and cross-talk or noise from adjacent muscles. To minimize these limitations and ensure data accuracy, all surface EMG measurements were conducted by a researcher with 8 years of experience in electromyographic research and extensive knowledge of muscle anatomy and physiology.

Surface EMG (TeleMyo DTS, Noraxon Inc., Scottsdale, AZ, USA) was used to measure the activity of the AD, PM, and PD muscles. The TeleMyo DTS directly transmits myoelectric data from the electrodes to a belt-worn receiver. EMG electrode placement for each muscle followed established protocols from previous studies [4,31]. For the AD, electrodes were placed 4 cm below the clavicle on the anterior aspect of the humerus. For the PM, electrodes were placed 2 cm medial to the axillary fold, toward the sternum. For the PD, electrodes were placed 2 cm below the lateral border of the scapular spine and angled obliquely toward the humerus. Before attaching electrodes, the skin was shaved and then cleaned with alcohol-soaked paper cotton. The EMG data were acquired at a sampling rate of 1000 Hz. Signal preprocessing included band-pass filtering using a finite impulse response filter (40–250 Hz), a 60 Hz notch filter to eliminate power line interference, and an infinite impulse response filter for additional noise suppression. Finally, the signals were full-wave rectified to prepare for subsequent analysis.

For signal normalization, the root mean square (RMS) values were calculated from 5 s maximal voluntary isometric contractions (MVICs) performed three times for each muscle. For the AD test, participants were seated in a short sitting position with their arms at their sides, elbows slightly flexed, and forearms pronated. They then flexed the shoulder to 90° without rotation or horizontal movement. Scapular abduction and upward rotation were permitted during the movement. Manual resistance was applied by the examiner, with one hand positioned over the participant’s distal humerus just proximal to the elbow, while the other hand stabilized the shoulder [32]. For the PM test, participants were positioned in a supine position with the shoulder abducted to 90° and the elbow flexed to 90°. They then performed shoulder horizontal adduction through the available range of motion. Manual resistance was applied by the examiner, with one hand positioned around the participant’s forearm just proximal to the wrist [32]. For the PD test, participants were seated in a short sitting position with the shoulder abducted to 90° and the elbow flexed to 90°. Manual resistance was applied by the examiner, with one hand positioned around the participant’s wrist, while the other hand supported the elbow to provide counterpressure at the end of the range of motion [32]. Each contraction was sustained maximally for 5 s. For data analysis, the middle 3 s of each MVIC were used, excluding the first and last second to eliminate transitional artifacts. Each test was repeated three times, with a 2 min rest between repetitions to minimize muscular fatigue. The examiner carefully monitored the participants during the isometric contractions to prevent any compensatory movements. EMG data from each trial were normalized to the RMS value obtained from the MVIC and expressed as a percentage of MVIC (%MVIC). The mean %MVIC across the three trials was used for statistical analysis [31].

### 2.4. Radiography

Radiography is a practical imaging modality for visualizing high-density structures such as bones. While it is not suitable for evaluating soft tissues like muscles, ligaments, or tendons, it is highly effective for assessing bony structures. In this study, radiographic imaging was employed to measure the displacement distance of the humeral head relative to the glenoid labrum before and after internal rotation of the shoulder. However, radiography involves exposure to ionizing radiation, albeit at a low level, and this exposure can accumulate over time. Therefore, prior to the imaging procedure, all participants were thoroughly informed about the purpose of the radiographic examination, the duration and frequency of imaging, and the potential radiation exposure. Written informed consent was obtained from each participant. All radiographic imaging was performed by a licensed radiologic technologist with five years of clinical experience.

Radiographic imaging (Accuray D5, DK Medical Systems Co. Ltd., Seoul, Republic of Korea) was performed to measure the displacement of the glenohumeral joint axis during the shoulder internal rotation exercises. All radiographs were acquired by the same radiologist to ensure consistency in image acquisition. A radiographic grid was positioned over the participant’s glenohumeral joint by the radiologist, and images were acquired both before and after the shoulder internal rotation. The center of joint rotation was defined as the midpoint of the line connecting the greater and lesser tubercles of the humeral head. Displacement of the center of rotation was determined by measuring the positional shift of the defined midpoint in a direction perpendicular to the line connecting the superior and inferior margins of the glenoid fossa. The magnitude of displacement was calculated by subtracting the vertical position of the joint center before internal rotation from its position after internal rotation [28].

### 2.5. Procedure

**Figure 1:**
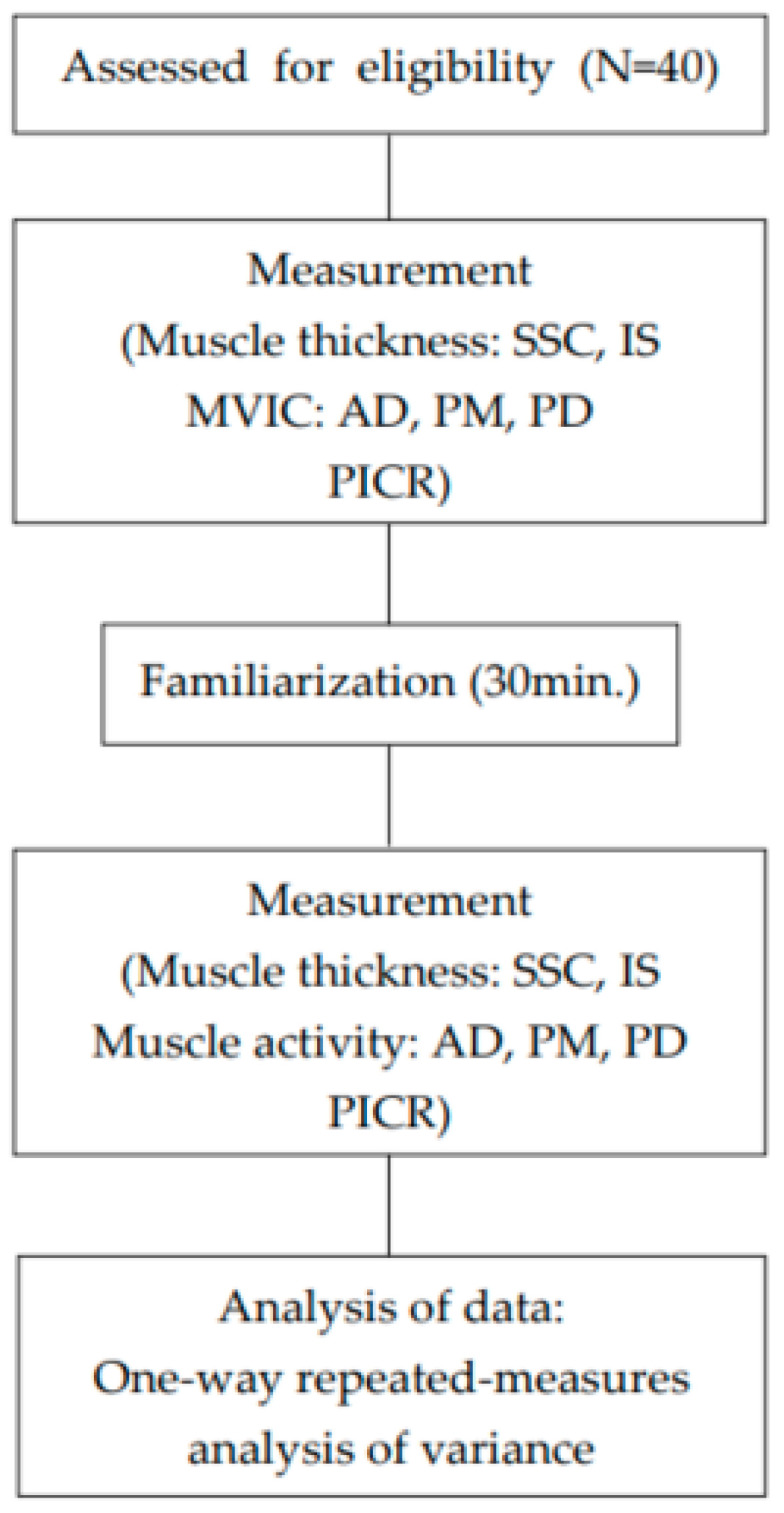
Experimental flowchart.

The experimental procedure for this study is outlined below (Figure 1).

**Figure 2:**
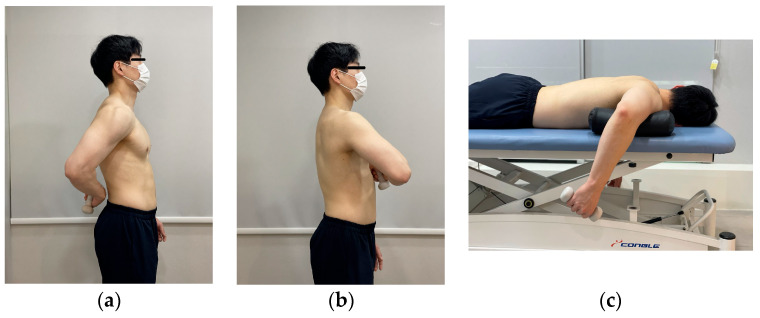
The three exercises: (**a**) Lift Off; (**b**) Belly Press; and (**c**) Prone Wiper.

This study was aimed to identify the most effective exercise for selectively strengthening the SSC, which functions as a stabilizer during shoulder internal rotation. All exercises were performed using the participants’ dominant arm, identified as the arm typically used for eating and writing. The three exercises—Lift Off, Belly Press, and Prone Wiper (Figure 2)—were performed in randomly assigned order. Before exercise familiarization, the examiner measured the resting muscle thickness of the SSC and IS, MVIC of the AD, PM, and PD, and PICR for each participant. The participants then practiced for 30 min to become familiar with maintaining a 5 s isometric contraction for each of the three rotation exercises. After completing the familiarization session, the participants rested for 15 min prior to the measurement phase, which was performed using a 1 kg dumbbell. Each participant completed three trials of each exercise with a 1 min rest between trials and a 3 min rest between exercises to minimize muscle fatigue [31]. Potential confounding variables such as participants’ prior experience and initial muscle strength were minimized by strictly adhering to the inclusion criteria at the beginning of this study. Additionally, to control for confounding factors related to individual differences in technical performance, such as muscle fatigue, rest periods were carefully standardized and strictly followed throughout the experimental procedure.

For the Lift Off, participants stood upright and placed the dorsum of the tested hand on the midpoint of the lumbar spine, assuming a position of shoulder internal rotation. Upon a verbal cue from the examiner, participants lifted the hand away from the spine as far as possible, performing additional internal rotation while maintaining the shoulder in a fixed position. The lifted position was held for 5 s and then returned to the starting position following a second verbal cue [4,14]. For the Belly Press, participants stood upright with the palm of the tested hand placed just below the xiphoid process and the elbows aligned horizontally, maintaining the trunk in the sagittal plane. Upon a verbal cue from the examiner, participants pressed the abdomen with the palm while keeping the shoulder stabilized and extended the elbow forward away from the trunk as far as possible. The position, which reflects shoulder internal rotation, was held for 5 s and then released to the starting posture following a second verbal cue [4,14]. For the Prone Wiper, participants were positioned prone with the shoulders abducted to 90° in the horizontal plane and the elbows flexed to 90°. Upon a verbal cue from the examiner, participants performed maximal shoulder internal rotation while maintaining the shoulder in a stabilized position. The internally rotated position was held for 5 s, after which participants returned to the starting position following a second verbal cue [33].

### 2.6. Statistical Analysis

A one-way repeated-measures analysis of variance (ANOVA) was conducted to compare the changes in muscle activity, muscle thickness, and PICR among the three exercises. Post hoc comparisons were performed using Bonferroni adjustment. A value of *p* < 0.05 was considered to indicate statistical significance. Effect sizes (Cohen’s d) were calculated to compare standardized mean differences among exercise conditions. Effect sizes were interpreted as small (0.20), medium (0.50), or large (0.80) based on Cohen’s conventional thresholds. Statistical analyses were conducted using SPSS version 18.0 for Windows (SPSS Inc., Chicago, IL, USA).

## 3. Results

### 3.1. General Characteristics of the Participants

The baseline characteristics of the participants are summarized below (Table 1).

### 3.2. Muscle Thickness

**Figure 3:**
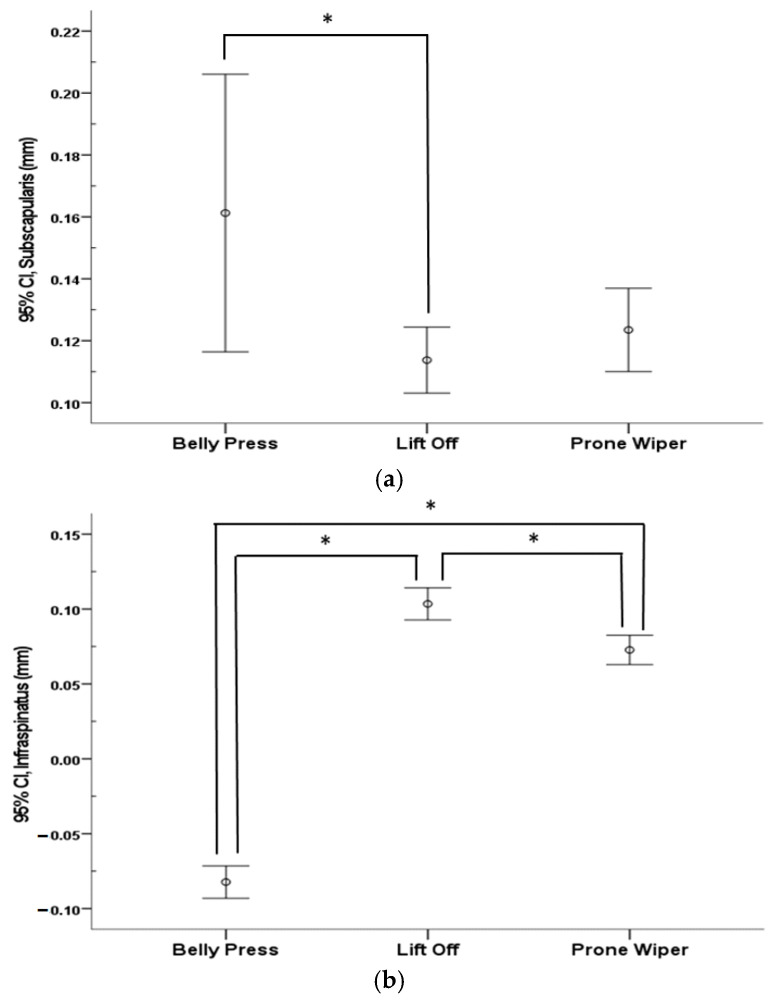
Comparison of muscle thickness among the three different exercises: (**a**) Subscapularis and (**b**) Infraspinatus. * indicates the difference between exercises.

A significant decrease in IS thickness during shoulder internal rotation was observed only in the Belly Press condition (*p* < 0.05). Except for this case, the thickness of all measured muscles significantly increased following shoulder internal rotation across all exercise conditions (*p* < 0.05) (Table 2). When comparing the three exercises, most changes in the muscle thickness of the SSC and IS showed significant differences (*p* < 0.05). A significant difference in SSC thickness was found between the Belly Press and Lift Off (*p* < 0.05), whereas no significant differences were observed between the Belly Press and Prone Wiper or between the Lift Off and Prone Wiper. Changes in IS thickness showed significant differences among all three exercises (*p* < 0.05) (Figure 3).

### 3.3. Muscle Activity

**Figure 4:**
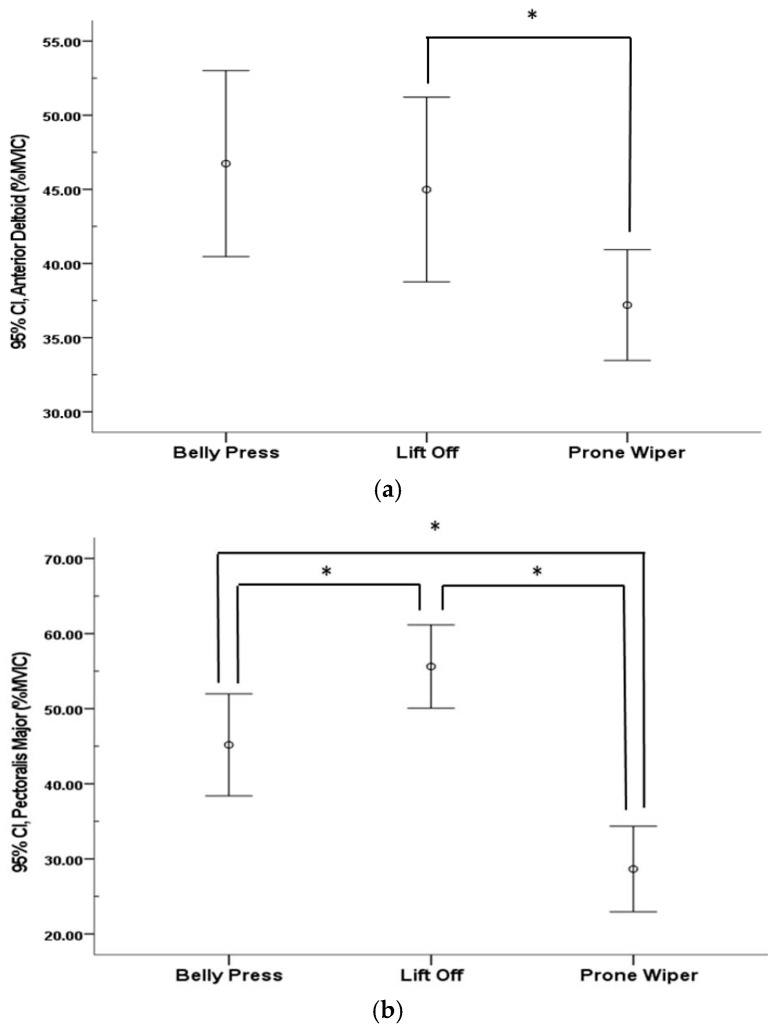

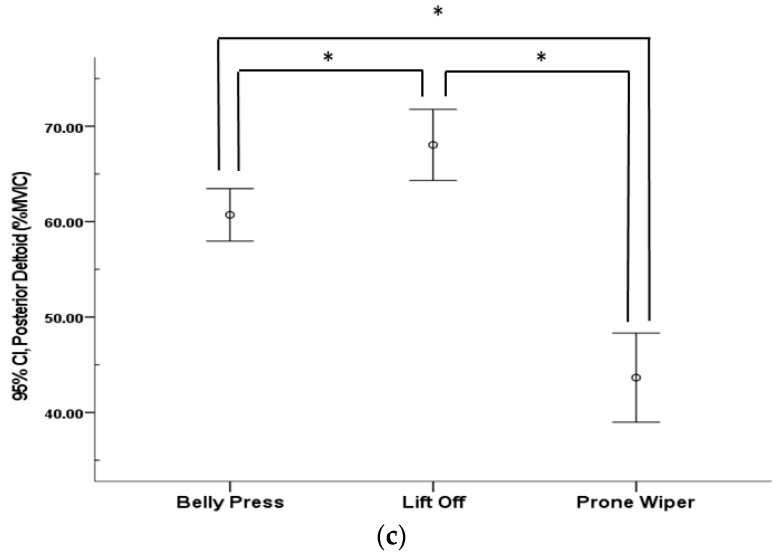
Comparison of muscle activity among the three different exercises: (**a**) anterior deltoid; (**b**) pectoralis major; and (**c**) posterior deltoid. * indicates the difference between exercises.

Muscle activity of the AD, PM, and PD significantly increased following shoulder internal rotation across all exercises (*p* < 0.05) (Table 3). A significant difference in AD activity was observed between the Belly Press and Prone Wiper (*p* < 0.05), whereas no significant differences were found in the other pairwise comparisons. In contrast, changes in PM and PD activity showed significant differences among all three exercises (*p* < 0.05) (Figure 4).

### 3.4. PICR

**Figure 5:**
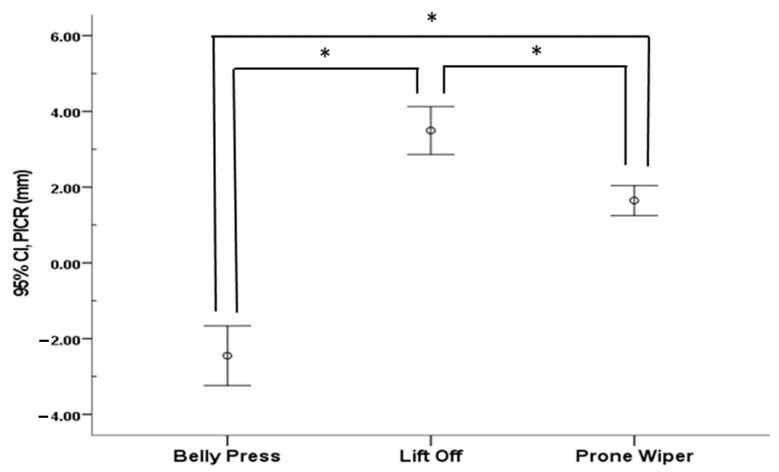
Comparison of path of the center of rotation among the three different exercises. Abbreviation: PICR, path of the instantaneous center of rotation. * indicates the difference between exercises.

The PICR significantly decreased during the Belly Press (*p* < 0.05), whereas it significantly increased following movement in both the Lift Off and Prone Wiper (*p* < 0.05) (Table 4). Comparative analysis revealed significant differences in PICR changes in the glenohumeral joint across all three exercises (*p* < 0.05) (Figure 5).

## 4. Discussion

The importance of the SSC in the treatment and management of RC injuries is well established. This study aimed to determine which exercise most effectively targets the SSC and to provide evidence for its use. To this end, we compared three exercises that are commonly used to improve shoulder internal rotation function: the Lift Off, the Belly Press, and the Prone Wiper. We then compared their effects on muscle thickness, muscle activity, and PICR changes.

Previous studies have shown that the IS functions as a stabilizer by generating a compressive force to maintain the humeral head within the glenoid fossa during shoulder abduction and external rotation [31]. The SSC also acts as a stabilizer by pulling the humeral head inferiorly to prevent superior translation during abduction and by resisting anterior translation during shoulder extension, thereby helping to center the humeral head in the glenoid fossa [4]. The SSC and IS, together with the supraspinatus, function as a force couple to counteract the superior translation of the humeral head induced by the deltoid, thereby maintaining joint centration within the glenoid cavity. In patients with shoulder impingement, however, increased activation of the middle deltoid—which contributes to superior humeral head migration—has been observed. Moreover, at the initiation of arm movement, when shear forces generated by the deltoid are at their peak, coactivation between the SSC–IS, supraspinatus–IS, and SSC–supraspinatus appears to be suppressed [34]. In a study on post-operative rehabilitation exercises for patients with RC injuries, Sgroi et al. (2018) found that prone external rotation with the shoulder abducted to 90° produced the greatest activation of the IS [16]. Additionally, internal rotation performed in a standing position with the shoulder abducted to 90° was found to elicit greater activation of the supraspinatus, IS, and SSC [16]. The observed increase in thickness in both the SSC and IS during the Lift Off and Prone Wiper exercises may reflect the coordinated action of these muscles functioning as stabilizers to maintain humeral head positioning within the glenoid fossa [35]. Another study compared the effects of various internal shoulder rotation exercises—including the Lift off and Belly Press—on strengthening the SSC and found that the SSC was strengthened to a similar extent regardless of the exercise posture [4]. However, the study also reported a significant reduction in the activation of other rotator muscles, with the exception of the SSC, during the Belly Press. This aligns closely with our findings, where the Belly Press significantly increased SSC thickness while decreasing IS thickness. Given the antagonistic relationship between the SSC, an internal rotator, and the IS, an external rotator, activation of one muscle may inhibit the activity of the other. [36]. Therefore, the significant decrease in IS thickness observed during the Belly Press may have facilitated more efficient SSC activation, as reflected by the greater increase in SSC thickness compared to the others.

The muscle activity of the AD, PM, and PD increased significantly across all exercises. The AD and PD function as a force coupled with the RC, contributing to shoulder elevation and rotation. Additionally, the PM contributes to shoulder elevation and abduction through the coordination with the latissimus dorsi and teres major muscles [37]. The SSC acts synergistically with these muscles to stabilize the humeral head inferiorly during shoulder elevation and abduction. Proper functioning of the scapular stabilizer muscles is critical for maintaining the center of rotation of the glenohumeral joint. Achieving an optimal balance between mobility and functional stability during shoulder rotation is essential for the effective distribution of the substantial forces acting on the shoulder joint [37]. Moradi et al. (2020) reported that, following an exercise intervention using elastic bands in adult male volleyball players with internal rotation deficits of the shoulder, there were significant increases in shoulder internal rotation range of motion, concentric and eccentric muscle strength, as well as in the muscle activation of the AD, PM, and PD [38]. Yu et al. (2023) compared the activation of the IS and PD during shoulder external rotation exercises with and without biofeedback and demonstrated that biofeedback enhanced the effective activation of the IS compared to exercises performed without it [39]. This contrasts with the present study, in which no biofeedback was applied, and suggests that the use of biofeedback may be necessary in future research aimed at selectively strengthening the SSC. Reinold et al. (2007) found that PD activation was significantly higher in the internally rotated empty-can position compared to the externally rotated full-can position [40]. Malanga et al. (1996) also reported that AD and PM activation significantly increased in the Jobe position, which involves internal rotation of the shoulder [1]. In addition, Kelly et al. (1996), in a manual muscle testing study of the RC, observed that internal rotation led to increased activation of the IS and PD [41]. These findings support the present results, which showed increased activation of the AD, PM, and PD across all three internal rotation exercises, along with increased IS thickness during the Lift Off and Prone Wiper.

Significant differences were also found in PICR across the three exercises. PICR increased during the Lift Off and Prone Wiper, whereas it decreased during the Belly Press. Variations in PICR during joint movement reflect shifts in the joint’s rotational center, and minimizing these shifts is essential for maintaining stable and normal joint mechanics [3]. During shoulder movement, the RC plays a key role in centering the humeral head within the glenoid fossa and generating compressive forces that help stabilize the joint’s center of rotation [42]. A previous study reported that the force couple generated by the RC muscles constrains the humeral head’s position of PICR to within ±1 mm relative to the glenoid fossa during abduction to 90° in the scapular plane from a resting position [28]. Furthermore, another study demonstrated that minimizing PICR deviations through activation of the IS enabled the deltoid muscle to produce high torque in a stable manner, thereby facilitating normal external rotation of the shoulder [27]. In this study, the SSC thickness increased across all exercises, with the greatest increase observed during the Belly Press. In contrast, the IS thickness decreased only during the role of preventing functional disorders such as impingement syndrome by centering the humeral head and limiting its forward and upward translation during shoulder movement [43,44]. Depending on shoulder posture, the IS contributes to the posterior of the Belly Press, while it increases with the other exercises. The SSC plays critical shoulder stability by reinforcing posterior structures and preventing humeral head subluxation during internal rotation [43]. It is hypothesized that the increased activity of the SSC—functioning both as a stabilizer and a primary mover in internal rotation—combined with reduced IS activity, its antagonist, enabled the AD and PM to generate torque more efficiently and with greater stability during the Belly Press. Consequently, a reduction in PICR distance was observed during this exercise, as confirmed by radiographic imaging—contrasting with the increases observed during the other exercises.

The SSC contributes to maintaining the center of rotation of the glenohumeral joint by counteracting the superior translation of the humeral head, particularly during dynamic shoulder movements such as abduction. When exercises aimed at selectively strengthening the SSC are properly performed, the muscle responds to the upward force exerted by the deltoid by generating a downward force on the humeral head. In the present study, the Belly Press was the only one to produce a reduction in the PICR by approximately 2.44 mm, indicating that it may be the most effective exercise for selectively activating the SSC. Unlike previous studies that initiated internal rotation movements from a resting position, this study began each movement from a preparatory posture specific to the exercise being performed [28]. This difference in starting position may account for the discrepancy in PICR values compared to the ±1 mm range of humeral head migration reported in earlier research. Future studies should investigate changes in muscle activation and PICR during the movement from a stable resting posture to the preparatory positions of each exercise in order to further validate and refine exercise selection for the targeted strengthening of the SSC.

The three exercises addressed in this study, including the Belly Press, can be readily applied in the assessment of patients with shoulder dysfunction. These exercises offer practical advantages, as they require minimal space and incur low costs. However, in order to perform these exercises correctly, an accurate clinical assessment of the patient’s symptoms and physical capabilities is essential. Clinicians must guide patients to perform the exercises within a safe and tolerable range, while carefully observing and preventing potential compensatory movements such as scapular elevation or spinal rotation, which may commonly occur during execution. Regular follow-up with the patient is also necessary for clinicians to monitor progress, adjust the exercise difficulty according to the patient’s ability, and encourage consistent adherence to the program in daily life.

This study has some limitations. First, all participants were healthy adults, which limits the generalizability of the findings to clinical populations with shoulder pathologies. Future research should include individuals presenting with shoulder dysfunction to determine whether the observed neuromuscular responses are consistent in symptomatic populations. Second, as the participants were limited to individuals in their late 30s, it is also difficult to generalize the findings of this study to all age groups. Third, the relatively small sample size may reduce the statistical power and restrict the external validity of the findings. Studies with larger and more diverse samples including participants across various age groups are needed to validate and extend these results. Fourth, a one-group pretest–posttest design without a control group was used in this study, which may have been vulnerable to external factors such as time effects or expectancy effects. So, it is difficult to establish causal relationships, and the internal validity of the findings may be limited. Future studies should adopt designs such as randomized controlled trials to enhance internal validity and reliability. Finally, the cross-sectional design of this study only allowed for the assessment of immediate, short-term effects. As such, it remains unclear whether the observed changes in muscle activity and PICR are sustained over time. Longitudinal studies are warranted to investigate the long-term efficacy and clinical relevance of these exercises in both healthy individuals and patient populations.

## 5. Conclusions

The Belly Press uniquely resulted in a significant increase in SSC thickness, a concurrent decrease in IS thickness, and a reduction in the PICR. Our results suggest that the Belly Press is the most effective of the three exercises in selectively activating the SSC while enhancing joint stability through minimized PICR distance. These findings may assist clinicians in designing more effective exercise programs. And, as noted above as a limitation, the findings of this study are preliminary and should be validated through future research employing more rigorous and robust designs to confirm their clinical efficacy.

## Figures and Tables

**Table 1:** General characteristics of the participants (N = 40).

Characteristics	Age (year)	Height (cm)	Weight (kg)
Male (N = 20)	39.9 ± 3.05 ^1^	170.53 ± 5.41	73.52 ± 9.72
Female (N = 20)	38.1 ± 3.38	162.35 ± 5.30	57.59 ± 6.14
Total	38.95 ± 3.32	166.44 ± 6.72	65.55 ± 11.38

^1^ Mean ± SD.

**Table 2:** Change of muscle thickness during movement in each exercise (N = 40) (unit: mm).

		Pre- Movement	Post-Movement	diff	t	*p*	95% CI	Effect Size
Belly Press	SSC	0.36 ± 0.11 ^1^	0.52 ± 0.21	0.16 ± 0.14	−7.27	0.000 *	[0.12, 0.21]	0.95
	IS	0.38 ± 0.08	0.3 ± 0.09	−0.08 ± 0.03	15.36	0.000 *	[−0.07, −0.09]	0.93
Lift Off	SSC	0.31 ± 0.08	0.43 ± 0.09	0.11 ± 0.03	−21.58	0.000 *	[0.09, 0.12]	1.43
	IS	0.31 ± 0.08	0.41 ± 0.09	0.10 ± 0.03	−19.5	0.000 *	[0.09, 0.11]	1.17
Prone Wiper	SSC	0.39 ± 0.11	0.51 ± 0.12	0.12 ± 0.04	−18.55	0.000 *	[0.11, 0.14]	1.09
	IS	0.21 ± 0.50	0.29 ± 0.05	0.07 ± 0.03	−15.02	0.000 *	[0.06, 0.08]	0.31

SSC, Subscapularis; IS, Infraspinatus; diff, difference. ^1^ Mean ± SD, * *p* < 0.05.

**Table 3:** Change in muscle activity during movement in each exercise (N = 40) (unit: %MVIC).

		Pre- Movement	Post-Movement	diff	t	p	95% CI	Effect Size
Belly Press	AD	8.58 ± 3.57 ^1^	55.32 ± 20.71	46.74 ± 3.10	−15.05	0.000 *	[41.61, 54.66]	3.14
	PM	13.38 ± 8.73	58.56 ± 23.77	45.18 ± 21.22	−13.46	0.000 *	[38.95, 52.76]	2.52
	PD	14.76 ± 7.14	75.48 ± 11.11	60.72 ± 8.62	−44.55	0.000 *	[57.65, 65.57]	6.50
Lift Off	AD	13.65 ± 7.13	58.64 ± 22.06	44.98 ± 3.07	−14.61	0.000 *	[38.24, 49.54]	2.74
	PM	15.01 ± 7.22	70.63 ± 15.84	55.62 ± 11.62	−20.25	0.000 *	[48.47, 61.39]	4.50
	PD	13.15 ± 6.93	81.20 ± 9.11	68.04 ± 11.62	−37.01	0.000 *	[64.16, 71.94]	8.40
Prone Wiper	AD	27.84 ± 9.75	65.03 ± 12.65	37.19 ± 11.67	−20.15	0.000 *	[32.89, 41.27]	2.60
	PM	26.66 ± 11.14	55.31 ± 22.12	28.65 ± 17.82	−10.16	0.000 *	[22.23, 33.38]	2.51
	PD	16.81 ± 8.84	60.46 ± 15.95	43.66 ± 14.61	−18.90	0.000 *	[39.33, 47.99]	4.78

AD, anterior deltoid; PM, pectoralis major; PD, posterior deltoid; diff, difference. ^1^ Mean ± SD, * *p* < 0.05.

**Table 4:** Displacement of center of rotation during movement in each exercise (N = 40) (unit: mm).

		Pre- Movement	Post-Movement	diff	t	*p*	95% CI	Effect Size
ICR	Belly Press	12.58 ± 4.39 ^1^	10.13 ± 3.84	−2.44 ± 1.26	6.27	0.000 *	[−3.24, −1.53]	0.59
	Lift Off	17.94 ± 2.8	21.44 ± 0.09	3.49 ± 1.97	−11.19	0.000 *	[2.83, 4.39]	0.21
	Prone Wiper	12.01 ± 4.67	13.66 ± 4.94	1.64 ± 1.24	−8.39	0.002 *	[1.17, 2.10]	0.16

ICR, instantaneous center of rotation; diff, difference. ^1^ Mean ± SD, * *p* < 0.05.

## Data Availability

All data related to this study are included in this article.
